# Does formaldehyde have a causal association with nasopharyngeal cancer and leukaemia?

**DOI:** 10.1186/s40557-018-0218-z

**Published:** 2018-01-31

**Authors:** Soon-Chan Kwon, Inah Kim, Jaechul Song, Jungsun Park

**Affiliations:** 1 Department of Occupational and Environmental Medicine, College of Medicine Soonchunhyang University, Cheonan, Republic of Korea; 2 Department of Occupational and Environmental Medicine, College of Medicine, Hanyang University, 222 wangshimni-ro, Seoul, 04763 Republic of Korea; 3 Department of Occupational Health, Catholic University of Daegu, Daegu, Republic of Korea

**Keywords:** Formaldehyde, Nasopharyngeal neoplasm, Leukemia, Workers’ compensation, Occupational diseases

## Abstract

**Background:**

The South Korean criteria for occupational diseases were amended in July 2013. These criteria included formaldehyde as a newly defined occupational carcinogen, based on cases of “leukemia or nasopharyngeal cancer caused by formaldehyde exposure”. This inclusion was based on the Internal Agency for Research on Cancer classification, which classified formaldehyde as definite human carcinogen for nasopharyngeal cancer in 2004 and leukemia in 2012.

**Methods:**

We reviewed reports regarding the causal relationship between occupational exposure to formaldehyde in Korea and the development of these cancers, in order to determine whether these cases were work-related.

**Results:**

Previous reports regarding excess mortality from nasopharyngeal cancer caused by formaldehyde exposure seemed to be influenced by excess mortality from a single plant. The recent meta-risk for nasopharyngeal cancer was significantly increased in case-control studies, but was null for cohort studies (excluding unexplained clusters of nasopharyngeal cancers). A recent analysis of the largest industrial cohort revealed elevated risks of both leukemia and Hodgkin lymphoma at the peak formaldehyde exposure, and both cancers exhibited significant dose-response relationships. A nested case-control study of embalmers revealed that mortality from myeloid leukemia increased significantly with increasing numbers of embalms and with increasing formaldehyde exposure. The recent meta-risks for all leukemia and myeloid leukemia increased significantly. In South Korea, a few cases were considered occupational cancers as a result of mixed exposures to various chemicals (e.g., benzene), although no cases were compensated for formaldehyde exposure. The peak formaldehyde exposure levels in Korea were 2.70–14.8 ppm in a small number of specialized studies, which considered anatomy students, endoscopy employees who handled biopsy specimens, and manufacturing workers who were exposed to high temperatures.

**Conclusion:**

Additional evidence is needed to confirm the relationship between formaldehyde exposure and nasopharyngeal cancer. All lymphohematopoietic malignancies, including leukemia, should be considered in cases with occupational formaldehyde exposure.

## Background

The South Korean Schedule for the Enforcement Decree of the Industrial Accident Compensation Act was amended in July 2013 to provide specific criteria for the recognition of occupational diseases, including occupational cancer. This amendment increased the official recognition from 9 cancers and 9 carcinogens to 21 cancers and 23 carcinogens. For example, the amendment formally recognized “leukemia and nasopharyngeal cancer [NPC] caused by formaldehyde [formaldehyde] exposure” [1, 2]. However, there are few detailed scientific reviews that have considered the relationship between formaldehyde exposure and leukemia in Korea.

The International Agency for Research on Cancer (IARC) officially classified formaldehyde as a “definite human carcinogen” for NPC in 2004 and for leukemia in 2012 [3, 4]. Other authorities have also acknowledged the possibly carcinogenic role of formaldehyde, including the American Environmental Protection Agency (EPA), the European Union Occupational Disease Classification, Labelling and Packaging of Substances and Mixtures (EU CLP) guidelines, and the American Conference of Governmental Industrial Hygienists (ACGIH) [5–7]. However, the 2010 amendment of the International Labor Organization (ILO) guidelines did not reach a consensus regarding whether occupational formaldehyde exposure was directly linked to NPC or leukemia [8]. Nevertheless, formaldehyde-related cancers are included in the lists of recognized occupational diseases in France, Denmark, Taiwan, and Malaysia [9–11].

Given the international trend towards recognizing formaldehyde-related occupational disease, and the absence of Korean reviews, we reviewed epidemiological studies and other evidence from Korea. We also present points for consideration during the process of determining whether formaldehyde-related NPC and leukemia should be considered work-related.

## Methods

We initially reviewed various scientific papers, including many epidemiological studies, regarding the causal relationship between formaldehyde exposure and cancer. Second, we reviewed various scientific papers, industrial reports, occupational exposure level reports, population data, and task force reports regarding exposure in Korean. Third, we reviewed various reports and epidemiological studies (including cohort studies, case-control studies, meta-analyses, reviews, and experimental studies) regarding the carcinogenicity of formaldehyde. Fourth, we investigate the national regulations regarding officially recognized occupational diseases and the international classifications for carcinogenicity. Finally, we considered the issues that could influence or determine the causal relationship between occupational exposure and cancer.

## Results

### Use and exposure in South Korea

Formaldehyde is mainly used in the production of various resins, although it is also used extensively as an intermediate during the manufacture of various industrial chemicals and directly as an aqueous disinfectant [3, 4]. The highest average exposures (2–5 ppm; 2.5–6.1 mg/m^3^) were measured during furniture and floor varnishing, textile finishing, fur treatment, in the garment industry, and in certain jobs at manufactured board mills and foundries. Short-term exposures to high levels (≥3 ppm; ≥3.7 mg/m^3^) have been reported for embalmers and pathologists [3].

In South Korea, employees who are exposed to formaldehyde have regular mandatory health examinations, which are mandatory for workers who are exposed to workplace hazards. Approximately 18,000 employees had formaldehyde-related health examinations during 2008, which accounted for 2.07% of all specific health examinations. Almost all specific health examinations were performed for people who worked in the manufacturing industry or in health and social work activities (Table 1).

**Table 1:** Number of workers examined special health check for formaldehyde by types of industries and years in Korea, 2001–2010

Industry	2001	2002	2003	2004	2005	2006	2007	2008	2009
D. Manufacturing	3359	4352	4912	5257	5527	7220	5371	9050	8305
P. Human health and social work activities	108	1072	1366	1915	2324	2845	2204	4643	4473
N. Public administration and defense; compulsory social security			13	197	1036	1323	1135	3128	1018
R. Membership organizations, repair and other personal services	32	69	125	176	255	222	94	461	131
M. Business facilities management and business support services	33	15	107	147	84	191	172	302	510
F. Construction	1	4	25	16	123	79	16	58	6
Others	12	24	111	113	126	91	69	83	96
Total	3545	5536	6659	7821	9475	11,971	9061	17,725	14,539

Industries are classified by the 8th Korean standard industrial classification

Source: Kim EA et al. [12]

Table 2 shows the formaldehyde exposure levels using a threshold limit value-time weighted average (TLV-TWA), based on national data regarding working environment measurements from 2002 to 2010. The highest number of samples was observed in industries that manufactured chemicals and chemical products, which was followed by the manufacture of motor vehicles, trailers and semitrailers, and non-furniture wood and cork products [12].

**Table 2:** Top 15 number of samples of measurement of concentration of work-environmental formaldehyde by sub-categories of industries in Korea, 2002–2010

Industry	No. of samples (A)	No. of detected samples (B)	Detection rate (B/A)	Geometric mean, ppm (range)
24. Manufacture of chemicals and chemical products	5370	4727	0.880	0.022 (0–25.598)
34. Manufacture of motor vehicles, trailers and semitrailers	3396	2929	0.862	0.011 (0–41.543)
20. Manufacture of wood products of wood and cork;except furniture	3075	2929	0.953	0.053 (0–62.479)
32. Manufacture of electrical components, audio, visual and communication equipment	2932	2608	0.889	0.020 (0–1.443)
27. Manufacture of basic metal products	2749	2414	0.878	0.015 (0–45.706)
17. Manufacture of textiles, except apparel	2409	2135	0.886	0.016 (0–16.800)
36. Manufacture of furniture and others	2227	2077	0.933	0.024 (0–4.152)
29. Manufacture of other machinery and equipment	1722	1286	0.747	0.014 (0–7.711)
85. Human health service	1630	1319	0.809	0.023 (0–25.300)
26. Manufacture of other non-metallic mineral products	1207	1075	0.891	0.034 (0–26.800)
28. Manufacture of fabricated metal products, except machinery and furniture	1020	922	0.904	0.023 (0–2.010)
25. Manufacture of rubber and plastic products	891	745	0.836	0.020 (0–0.990)
21. Manufacture of pulp, paper and paper products	739	632	0.855	0.013 (0–2.384)
31. Manufacture of electrical equipment	558	446	0.862	0.006 (0–32.900)
73. Scientific research and development	501	107	0.953	0.016 (0–1.500)

Industries are classified by the 8th Korean standard industrial classification

Source: Kim EA et al. [12]

Table 3 shows the results of the formaldehyde exposure levels based on results from academic reports in Korea. The maximum exposure level was 5.01 ppm in histological laboratories from nine general hospitals [13]. The maximum formaldehyde exposure level was 3.91 ppm among 80 students in a gross anatomy laboratory, which was sampled five times in four areas [14]. The maximum formaldehyde exposure level was 14.77 ppm among 48 workers in the endoscopy units of four general hospitals [15].

**Table 3:** Cross-sectional studies about workplace measurements of formaldehyde in Korea

Authors (Years)	Subjects and measurements	Formaldehyde concentration, ppm Geometric mean (range)
Park et al., (1998) [13]	19 workers in 9 histological laboratories in each hospitals. Personal and area sampling to assess TWA and STEL	TWA (8 h) Personal 0.31 (0.02–3.86) Area 0.61 (0.08–1.49). STEL (15 min) Personal 1.88 (0.59–5.01) Area 1.42 (0.31–4.24)
Park et al.,(2006) [14]	80 students in a gross anatomy laboratory in a college of medicine. Area sampling at 4 spots 5 times each	TWA (8 h) Area 1.51 (0.26–3.91)
Kim et al. (2009) [15]	48 workers in endoscopy units in 4 hospitals. Personal samplings to assess TWA and STEL.	TWA (8 h) Personal 0.056 (0.003–0.923) STEL (15 min) Personal 1.428 (0.103–14.773)
Lee et al., (2012) [16]	In curling and compounding process of two tire plants, 12 personal sampling to assess TWA.	TWA (8 h) Personal 0.029 (0.027–0.029) (highest within 4 spots)
Yoo et al.,(2014) [17]	Workers handle medium density fiberboard and veneer in a furniture manufacturing factory. Air concentration of formaldehyde handling wood glue and wet veneer at room temperature, 100 °C and 150 °C	(Handling wet veneer at 150 °C, 2.70 ppm)
Gu et al., (2014) [18]	62 nurses in 8 ambulatory care units in 2 hospitals. Personal samplings to assess TWA.	TWA (8 h) Personal 0.023 (0.001–0.258)

TWA, Time weighted average; STEL, Short-term exposure limit

The highest concentration of formaldehyde was 0.029 ppm in the compounding process of two tire manufacturing plants [16]. A furniture manufacturing factory had a formaldehyde concentration of 2.7 ppm when handling wet veneer at 150 °C [17]. The maximum formaldehyde exposure level was 0.258 ppm among 62 nurses in two university hospitals [18].

### Epidemiological studies

#### NPC

The main epidemiological results for NPC have been obtained from a National Cancer Institute (NCI) cohort that included 10 plants that produced or used formaldehyde. The results revealed a significantly increase risk of death because of NPC and dose-response relationships with both peak and cumulative formaldehyde exposures [19]. The strength of the associations weakened and the dose-response relationships for cumulative exposure levels disappeared after 10 years of follow-up [20]. Marsh et al. reported that this result was related to the effect of the first factory, and they reported that the excess death because of NPC was the result of a work history involving silversmithing or other metal processing [21, 22]. However, the IARC committee concluded that the effect modification based on silversmithing or other confounding could not explain the excess death because of NPC [4]. Another cohort study of a British chemical plant, an American clothing manufacturer, a Finish cancer registry, and an Italian plastic factory did not detect a significant risk of formaldehyde-related NPC, with the exception of an unexplained cluster of deaths because of NPC at plant 1 in the NCI cohort [23–28] (Table 4). Several case-control studies have also reported a significant relationship or dose-response relationship between the highest formaldehyde exposure and NPC [29–31]. However, we did not detect significant relationships in other studies [32–35] (Table 5). The results from a meta-analysis (excluding plant 1 of the NCI cohort) are shown in Table 6, and the meta-risk was 0.72 (95% confidence interval [CI]: 0.40–1.29) [36–38].

**Table 4:** Cohort studies of formaldehyde exposures and nasopharyngeal cancer

Authors (Years), Country	Cohort description Type of analysis (cohort size)	Exposure assessment	Results SMR or RR (95% CI)
Hauptmann et al.(2004), USA [19]	The cohort with 10 plants of manufacture of or using formaldehyde by the National Cancer Institute from 1966 to 1994; Standardized mortality (25,619 workers; 22,493 men, 3126 women) and Relative risks	Duration; quantitative estimates of cumulative, average and highest peak exposure	8 death, SMR 2.10 (1.05–4.21) Peak exposure (ppm) ≥4.0 RR 1.83 (*p*-trend < 0.001) Cumulative exposure (ppm-year) 1.5–< 5.5 RR 1.19, ≥5.5 RR 4.14 (*p*-trend = 0.025)
Freeman et al. (2013), USA [20]	Update of Hauptmann et al.(2004), 1966–2004	Duration; quantitative estimates of cumulative, average and highest peak exposure	9 death, SMR 1.84 (0.84–3.49); Peak Exposure (ppm) ≥4.0 RR 7.66 (0.94–62.34) (*p*-trend = 0.005)
Marsh et al. (2005), USA [21]	Computed SMRs and RRs for each of 10 study plants and by plant group (Plant 1 (*n* = 4261) vs. Plants 2–10 (*n* = 21,358)). 1966–1994	Duration; quantitative estimates of cumulative, average and highest peak exposure	Plant 1 6 deaths, SMR 10.32 (3.79–22.47) plant 2–10 2 deaths, SMR 0.65 (0.08–2.33)
Coggon et al. (2003), UK [23]	Chemical factories that used or produced Formaldehyde; Standardized mortality (14,014 men); 1941–2000	Level of exposure (background, low, moderate, high); among highly exposed, time period and duration of exposure	One death from nasopharyngeal cancer (2.0 expected)
Coggon et al. (2014), UK [24]	Update of Coggon et al., (2003). (14,008 men); 1941–2012	Level of exposure (background, low, moderate, high); among highly exposed, time period and duration of exposure	One death from nasopharyngeal cancer (1.7 expected)
Pinkerton et al. (2004), USA [25]	Garment industry; Standardized mortality (11,039 workers; 2015 men, 9024 women)	Duration, time since first exposure, year of first exposure	No death from nasopharyngeal cancer (0.96 expected)
Meyers et al. (2013), USA [26]	Garment industry; Standardized mortality (11,039 workers; 2015 men, 9024 women)	Duration, time since first exposure, year of first exposure	No death from nasopharyngeal cancer (0.96 expected)
Siew et al. (2012), Finland [27]	All Finnish men born between 1906 and 1945 and employed during 1970, Finnish Cancer Registry for cases of cancers of nasopharynx (*n* = 149) (*n* = 30,317). 1971–1995.	Estimation of exposure by Finnish Job Exposure Matrix	Any exposure to formaldehyde RR 0.87 (0.34–2.20) Any exposure to wood dust RR 0.66 (0.30–1.45)
Pira et al. (2012), Italia [28]	Workers of a laminated plastic factory in Piedmont, northern Italy. 2750 subjects (2227 men and 523 women) between 1947 and 2011, for at least 180 days. SMRs	None	No nasopharyngeal cancer was confirmed.

SMR, Standardized mortality ratio; RR, Relative risk

**Table 5:** Case-control studies of formaldehyde exposure and nasopharyngeal cancer

Authors (Years), Country	Characteristics of cases and controls	Exposure assessment	Exposure categories	OR (95% CI)
Marsh et al. (2007), USA [22]	7 incidental cases who died from nasopharyngeal cancer during 1945–2003; case was matched on exact age race, sex, and year of birth (±2 years) to four controls from the members of the cohort.	Evaluation of formaldehyde exposure while accounting for potential confounding or effect modification by smoking or external (non-Wallingford) employment.	Silver smithing	
			Never	1.0 (Ref)
			Ever	14.41 (1.30–757.8)
			*p*-trend	0.024
			Silver smithing or other metal work	
			Never	1.0 (Ref)
			Ever	7.31 (1.08–82.1)
			*p*-trend	0.047
Olsen et al. (1984), Denmark [32]	754 cases from the Danish Cancer Registry including 266 nasopharyngeal cancers; controls were 2465 patients with cancers of the colon, rectum, prostate and breast; frequency matched by sex, age (± 5 years) and year of diagnosis (± 5 years)	Record linkage with pension fund with compulsory membership; job title from Central Pension Registry; exposure assessed blindly as certain, probable, unlikely, unknown	Men	0.7(0.3–1.7)
			Women	2.6(0.3–21.9)
Vaughan et al. (1986), USA [33]	285 incidental cases identified by the local Cancer Surveillance System, aged 20–74 years, including 27 cases of cancer of nasopharynx; controls were 552 identified by random-digit dialing	Job–exposure linkage system based on industry and occupation, resulting in four categories: high, medium, low and background	Low exposure	1.2 (0.5–3.3)
			Medium or High	1.4 (0.4–4.7)
			Exposure years	
			1–9	1.2 (0.5–3.1)
			10+	1.6 (0.4–5.8)
			Exposure scores	
			5~ 19	0.9 (0.5–5.7)
			20+	2.1 (0.6–7.8)
Roush et al. (1987), USA [29]	173 nasopharyngeal cancers registered at the Connecticut Tumor Registry; Controls were 605 men who died during the same period, selected by random sampling without matching or stratification	Job title, industry, specific employment, year of employment, obtained from death certificates and city directories to determine occupation at 1, 10, 20, 25, 30, 40 and 50 years prior to death	Probably exposed for most of working	2.7 (1.1–6.6)
			life	1.2 (0.5–3.2)
			+ exposure > 20 years before death	2.9 (1.1–7.6)
			+ exposure to high level for some years	4.0 (1.3–12.3)
			+ exposure to high level > 20 years before death	
West et al. (1993), Philippines [30]	104 incidental cases (76 men, 28 women) histologically confirmed; 104 hospital controls matched for sex, age and hospital ward type and 101 community controls matched for sex, age and neighborhood	Occupational history; occupation classified as likely or unlikely to involve exposure to formaldehyde; duration of exposure; 10-year lag period; years since first exposure; age at start of exposure	Exposure year	
			< 15 years	2.7 (1.1–6.6)
			> 15 years	1.2 (0.5–3.2)
			< 15 years (10-year lag)	2.9 (1.1–7.6)
			> 15 years (10-year lag)	4.0 (1.3–12.3)
Vaughan et al. (2000), USA [31]	196 men and women from five cancer registries, aged 18–74 years controls were 244 population based selected by random digit dialing, and frequency matched by sex, cancer registry and age (5-year groups)	Structured telephone interviews; occupational exposures assessed by a job–exposure matrix	Duration (years)	
			1–5	0.9 (0.4–2.1)
			6–17	1.9 (0.9–4.4)
			> 18	2.7 (1.2–6.0)
			*p*-trend	0.014
			Cumulative exposure (ppm-years)	
			0.05–0.4	0.9 (0.4–2.0)
			0.4–1.10	1.8 (0.8–4.1)
			> 1.10	3.0 (1.3–6.6)
			*p*-trend	0.03
Armstrong et al. (2000), Malaysia [34]	282 histologically confirmed cases of nasopharyngeal carcinoma in Chinese men and women from four centers who had lived in the area for > 5 years;One Chinese control selected by multistage area sampling per case, matched by age and sex	Structured in-home interviews; Occupational exposures assessed by a job–exposure matrix	Any (unadjusted)	1.24 (0.67–2.32)
			Any (adjusted)	0.71 (0.34–1.41)
Hildesheim et al. (2001), Taipei [35]	375 histologically confirmed hospital cases (31% women), aged < 75 years; 325 community controls, individually matched on sex, age (5 years) and district of residence	Structured in person interviews; occupational exposures assessed by an industrial hygienist	Ever exposed	1.4 (0.93–2.2)
			Duration	
			1–10 years	1.3 (0.69–2.3)
			> 10 years	1.6 (0.91–2.9)
			*p*-trend	0.08

OR, Odds ratio

**Table 6:** Meta-analysis of formaldehyde exposure and nasopharyngeal cancer

Authors, (Years)	Studies	Overall OR or RR (95% CI)
Collins et al.,(1997) [36]	Cohort studies with reported expected deaths Industrial cohort studies Case-control studies All Studies	1.6 (0.8–3.0) 1.2 (0.4–2.5) 1.3 (0.9–2.1) 1.3 (1.2–1.5)
Bosetti et al. (2008) [37]	Hauptmann et al. 2004-plant 1 Hauptmann et al. 2004-plants 2–10 Industrial cohort studies	9.10 (4.09–20.26) 0.64 (0.16–2.56) 1.33 (0.69–2.56)
Bachand et al. (2010) [38]	Cohort studies (excluding single plant) Case control studies	0.72 (0.40–1.29) 1.22 (1.00–1.50)

### Lymphohematopoietic malignancies

Six of the seven mortality studies involving professional workers (e.g., embalmers, funeral directors, pathologists, and anatomists) revealed positive associations between formaldehyde exposure and lymphohematopoietic malignancies (LHM) [39–45] (Table 7). The NCI cohort compared deaths from 2004 and 1994, and found that the strength of association between formaldehyde exposure and death because of leukemia and myeloid leukemia was weakened. Furthermore, the peak-exposure category (≥4.0 ppm) exhibited dose-response relationships with LHM, myeloid leukemia, and Hodgkin lymphoma [46–48]. Three cohort studies failed to detect a significantly increased risk of death [23–26]. Three case-control studies of formaldehyde exposure and leukemia also failed to detect a significantly increased risk [49–51]. A nested case-control study of funeral professionals revealed that the risks of non-lymphoid LHM or myeloid leukemia increased with working experience [52] (Table 8). Table 9 shows results from a meta-analysis of the relationship between formaldehyde exposure and leukemia [37, 38, 53–55]. The risk estimate for all leukemia was 1.05 (95% CI: 0.93–1.20) when researchers included the recent NCI cohort and excluded proportionate mortality studies [38]. The meta-risks including the NCI cohort and American funeral industries were 1.53 (95% CI: 1.11–2.21) for all leukemia and 2.47 (95% CI:1.42–4.27) for myeloid leukemia [55]. Nevertheless, researchers have not reached a consensus regarding any causal association or dose-response relationship between formaldehyde exposure and LHM, including myeloid leukemia [56, 57]. However, there appears to be a causal association between formaldehyde exposure, and especially peak exposures of ≥4 ppm, and all LHM (including Hodgkin lymphoma but not leukemia).

**Table 7:** Cohort studies of formaldehyde exposures and exposures and lymphohematopoietic malignancies

Authors (Years), Country	Cohort description Type of analysis (cohort size)	Exposure assessment	Results SMR or PMR (95% CI)
Walrath et al. (1983), USA [39]	Embalmers and funeral directors, PMR/PCMR (1132 white men); 1925–80	Time since first license, age at first license	LHM 25 deaths PMR 1.21 Leukemia 12 deaths PCMR 1.19 Myeloid leukemia 6 deaths PCMR 1.5
Walrath et al. (1984), USA [40]	Embalmers, PMR/PCMR(1007 white men)	Duration	LHM 19 deaths PCMR 1.22 Leukemia 12 deaths PCMR 1.40 Myeloid leukemia 6 deaths PCMR 1.50
Levine et al. (1984), Canada [41]	Embalmers, SMR(1413 men); 1950–77	None	LHM 8 deaths SMR 1.23 (0.53–2.43) Leukemia 4 deaths SMR 1.60 (0.44–4.10)
Stroup et al. (1986), USA [42]	Anatomists, SMR (2239 men); 1925–79	Duration	LHM 18 deaths SMR 1.2 (0.7–2.0) Lymphoma 2 deaths SMR 0.7 (0.1–2.5) Leukemia 10 deaths SMR 1.5 (0.7–2.7) CML 3 deaths SMR 8.8 (1.8–25.5) Other lymphoma 6 deaths SMR 2.0 (0.7–4.4)
Logue et al. (1986), USA [43]	Pathologists, SMR (5585 men); 1962–77	None	LHM SMR 0.48 Leukemia SMR 1.06
Hayes et al. (1990), USA [44]	Embalmers/funeral directors, PMR(3649 white men, 397 non-white men); 1975–85	None	LHM 100 deaths PMR 1.31 (1.06–1.59) (White) 15 deaths PMR 2.41 (1.35–3.97) (Non-white) Myeloid leukemia 23 deaths PMR 1.61 (1.02–2.41) (White) 1 death PMR 1.06 (0.02–5.93) (Non-white) Other unspecified leukemia 17 deaths PMR 2.08 (1.21–3.34) (White) 3 deaths PMR 4.92 (1.01–14.36) (Non-white)
Hall et al. (1991), UK [45]	Pathologists; SMR; 4512 (data presented for 3872, 802 women, 3069 men); 1974–87	None	LHM 10 deaths SMR 1.44 (0.69–2.65) Hodgkin lymphoma 1 death SMR 1.21 (0.03–6.71) Leukemia 10 deaths SMR 1.5 (0.7–2)
Hauptmann et al. (2003), USA [46]	The cohort composed of 10 plants of manufacture of or using by the National Cancer Institute during 1966–1994; Standardized mortality (25,619 workers; 22,493 men, 3126 women) and Relative risks	Duration; quantitative estimates of cumulative, average and highest peak exposure	Peak exposure (ppm) LHM 2.0–3.9 RR 1.71 (1.14–2.58) ≥4 RR 1.87 (1.27–2.75) (*p*-trend, 0.002) Leukemia 2.0–3.9 RR 2.04 (1.04–4.01) ≥4 RR 2.46 (1.31–4.62) (*p*-trend, 0.004) Myeloid leukemia ≥4 RR 3.46 (1.27–9.43) (*p*-trend, 0.009)
Freeman et al. (2009), USA [47]	Update of Hauptmann et al.(2003); 1966–2004	Duration; quantitative estimates of cumulative, average and highest peak exposure	Peak exposure (ppm) LHM ≥4 RR 1.37 (1.03–1.81) (*p*-trend, 0.02) Hodgkin lymphoma 2.0–3.9 RR 3.30 (1.04–10.50) ≥4 RR 3.96 (1.31–12.02) (*p*-trend, 0.01)
Checkoway et al. (2015), USA [48]	Re-analyses of Freeman et al. (2009), Analysis of full cohort (*n* = 25,619) and workers employed 1 year or longer (*n* = 16,306); Cox proportional hazards analyses; 1966–2004	Duration; quantitative estimates of cumulative, average and highest peak exposure	Worked ≥1 year, Peak Exposure (ppm) Hodgkin lymphoma ≥2.0–*<* 4 HR 3.50 (1.06–11.56) ≥4 HR 5.13 (1.67–15.77) (*p*-trend, 0.003) All leukemia ≥2.0–*<* 4 HR 2.46 (1.29–4.67) ≥ HR 2.45 (1.32–4.52) (*p*-trend, 0.002)
Coggon et al. (2003), UK [23]	Garment industry; Standardized Mortality (11,039 workers), 1955–1998	Duration, time since first exposure, year of first exposure	LHM 59 deaths SMR 0.97 (0.74–1.26) Leukemia 24 deaths SMR 1.09 (0.70–1.62) Myeloid leukemia 15 death SMR 1.44 (0.80–2.37)
Coggon et al. (2014), UK [24]	Update of Pinkerton et al., (2004). (11,043 workers), 1955–2008		LHM 107 deaths SMR 1.11 (0.91–1.34) Leukemia 36 deaths SMR 1.04 (0.73–1.44) Myeloid leukemia 21 death SMR 1.28 (0.79–1.96)
Pinkerton et al. (2004), USA [25]	Chemical ctories that used or produced Formaldehyde; Standardized mortality (14,014 men); 1941–2000	Level of exposure (background, low, moderate, high); among highly exposed, time period and duration of exposure	Leukemia All subjects 31 deaths SMR 0.91 (0.62–1.29) High exposure 9 deaths SMR 0.71 (0.31–1.39)
Meyers et al. (2013), USA [26]	Update of Coggon et al., (2003). (14,008 men); 1941–2012	Level of exposure (background, low, moderate, high); among highly exposed, time period and duration of exposure	Leukemia All subjects 36 deaths SMR 1.02 (0.77–1.33) High exposure 13 deaths SMR 0.82 (0.44–1.41)

SMR, Standardized mortality ratio; LHM, Lymphohematopoietic malignancies

SMR, Standardized mortality ratio; PMR, Proportionate mortality ratio; PCMR, Proportionate cancer mortality ratio; LHM, Lymphohematopoietic malignancies; CML, Chronic myeloid leukemia

RR, Relative risk; LHM, Lymphohematopoietic malignancies; HR, Hazard ratio

**Table 8:** Case-control studies of formaldehyde exposure and lymphohemtopoietic malignancies

Authors (Years), Country	Characteristics of cases and controls	Exposure assessment	Exposure categories	OR (95% CI)
Lions et al. (1990), USA [49]	578 male cases of leukemia and 722 non-Hodgkin lymphoma; 1245 population-based controls	Lifetime occupational history	Ever employed in funeral home or	
			crematorium	
			Leukemia	2.1 (0.4–10.0)
			Non-Hodgkin lymphoma	3.2 (0.8–13.4)
Partanen et al. (1993), Finland [50]	Nested case control study, Cohort with workers in wood industry (*n* = 7307); Leukemia (*n* = 12), Hodgkin lymphoma (*n* = 4) and Non-Hodgkin lymphoma (*n* = 8); Matched by year of birth and vital status in 1983. Leukemia (n = 73), Hodgkin lymphoma (*n* = 21) and Non-Hodgkin lymphoma (*n* = 52)	Work history from company records complemented for cases only by interviews with plant personnel and questionnaires completed by subjects or next of kin; plant- and period-specific job–exposure matrix	Leukemia	
			≥ 3 ppm-months	1.40 (0.25–7.91)
			Hodgkin diseases	
			≥ 3 ppm-months	-
			Non-Hodgkin disease	
			≥ 3 ppm-months	4.24 (0.68–26.6)
Blair et al. (2001), USA [51]	513 white men, 30 ≥ years, from the Cancer Registry of Iowa and a surveillance network of hospitals in Minnesota; 132 acute myeloid, 46 chronic myeloid leukemia cases; 1087 controls by random-digit dialing, frequency matched by 5-year age group, vital status at time of interview and state of residence	Personal interviews including lifetime occupational history; formaldehyde assessed in a blinded shion in terms of probability and intensity, each on a 4-point scale based on job title and industry	Acute myeloid leukemia	
			Low-medium	0.9 (0.5–1.6)
			High	-
			Chronic myeloid leukemia	
			Low-medium	1.3 (0.6–3.1)
			High	2.9 (0.3–24.5)
Hauptmann et al. (2009), USA [52]	168 professionals in the funeral industry and died from lymphohematopoietic malignancies; 265 matched controls were randomly selected from individuals in the same industry whose deaths were attributed to other causes and were stratified to be similar to the case subjects with respect to data source, sex, and dates of birth and death (5-year intervals); 1960–86.	Life time work practices and exposures in the funeral industry, which were obtained by interviews with next of kin and coworkers, and to estimated levels of formaldehyde exposure.	Myeloid Leukemia	
			Embalming ever	11.2 (1.3–95.6)
			Duration of embalming (year)	
			> 0–20	5.0 (0.5–51.6)
			> 20–34	12.9 (1.4–117.1)
			> 34	13.6 (1.6–119.7)
			*p*-trend	0.020
			< 500 embalmings	1.0 (Ref.)
			Duration of embalming (year)	
			≤20	0.5 (0.1–2.9)
			> 20–34	3.2 (1.0–10.1)
			> 34	3.9 (1.2–12.5)
			*p*-trend	0.020

OR, Odds ratio

**Table 9:** Meta-analysis of formaldehyde exposure and lymphohemtopoietic malignancies

Authors, (Years)	Studies	Overall OR or RR (95% CI)
Collins et al. (2004) [53]	All studies for for leukemia Industrial workers Embalmers Pathologists and anatomist	1.1 (1.0–1.2) 0.9 (0.8–1.0) 1.6 (1.2–2.0) 1.4 (1.0–1.4)
Bosetti et al. (2008) [37]	All LHM	
	Industrial workers	0.85 (0.74–0.96)
	Professionals	1.31 (1.16–1.48)
	Leukemia	
	Industrial workers	0.90 (0.75–1.07)
	Professionals	1.39 (1.15–1.68)
Zhang et al. (2009) [54]	All LHM All leukemia Myeloid leukemia Hodgkin lymphoma Non-Hodgkin lymphoma Multiple myeloma	1.25 (1.12–1.39) 1.54 (1.18–2.00) 1.90 (1.31–2.76) 1.23 (0.67–2.29) 1.08 (0.86–1.35) 1.31 (1.02–1.67)
Bachand et al. (2010) [38]	All leukemia Professional/technical workers Industrial workers Myeloid leukemia Lymphoid leukemia	1.05 (0.93–1.20) 1.28 (0.98–1.66) 0.99 (0.86–1.15) 1.09 (0.84–1.40) 1.11 (0.81–1.52)
Schwik et al. (2010) [55]	All leukemia Professional workers Industry workers Healthy-worker effect adjusted Myeloid leukemia Healthy-worker effect adjusted	1.53 (1.11–2.21) 2.27 (1.15–4.45) 1.38 (0.96–1.99) 1.72 (1.18–2.51) 2.47 (1.42–4.27) 2.77 (1.39–5.52)

LHM, Lymphohematopoietic malignancies

### Biological plausibility

There is no clear carcinogenic mechanism regarding formaldehyde exposure and NPC or LHM. However, formaldehyde exposure can lead to the formation of DNA-protein crosslinks in vitro, as well as genotoxicity in human nasal cells and disruption of bone marrow stem cells, hematopoietic stem cells, circulating progenitor cells, and primitive pluripotent stem cells [58, 59]. Chromosomal aneuploidy in circulating myeloid progenitor cells has also been identified among healthy workers who were exposed to formaldehyde [60].

### Criteria for recognizing formaldehyde as an occupational carcinogen

The IARC classified formaldehyde as a definite human carcinogen (Group 1) for NPC in 2004 and leukemia (especially myeloid leukemia) in 2012. Formaldehyde was also classified as a suspected human carcinogen (Group 2A) for sino-nasal cancer in 2012 [3, 4]. The American National Toxicology Program (NTP) also classified formaldehyde as a ‘known human carcinogen’ in 2011 [61]. Furthermore, the EU CLP classified formaldehyde as a class 1B carcinogen, which indicates that the substance has presumed carcinogenic potential in humans, based on experimental animal data [6].

The ILO includes 20 carcinogens on its list of occupational cancers, although it does not define the related cancers. The tripartite commission of ILO included formaldehyde on its list of potential carcinogens, although formaldehyde was not included in the final list in 2009, as employers demanded a deeper review of the data [8, 9]. South Korea, France, Denmark, Malaysia, and Taiwan have clearly recognized the relationship between occupational cancer and formaldehyde exposure [9–11]. France also recognized that NPC could be caused by exposure to formaldehyde or its polymers in 2009 [10]. However, the list of recognized occupational diseases in Finland does not include formaldehyde-related cancers, although it was considered in the “Memorandum from the Occupational Cancer Working Group 2013” [62]. Moreover, the EU only recognizes a relationship between formaldehyde exposure and NPC, as there is insufficient epidemiological evidence regarding LHM [63].

### Compensation cases and considerations for approval

South Korea has not compensated any cases that were related to formaldehyde exposure itself, although some cases have been compensated after mixed exposures to other chemicals. In 2012, a 61-year-old man developed multiple myeloma after working at a poultry farm for 16 years and being exposed to agricultural chemicals (pesticides and/or organic solvents, such as formaldehyde), with an average estimated formaldehyde exposure level of 17.53 ppm [64]. A 43-year-old man was diagnosed with myelodysplastic syndrome after working in a furniture manufacturing factory for 22 years. In 2013, the man’s tasks involved cutting and fabricating medium-density fiberboard, as well as pasting and polishing veneer. He was exposed to benzene and formaldehyde (a TWA concentration of 0.312 ppm/8 h), which corresponded to a cumulative level of 6962–10,016 ppm·hour, and a cumulative benzene exposure of 1.88–11.25 ppm·year [65].

### Recognition criteria and consideration issues

Since 2013, the occupational disease criteria of the Enforcement Decree Industrial Accidents Compensation Insurance Act has included “leukemia or NPC caused by formaldehyde exposure” [2]. However, there is little evidence regarding the cumulative exposure level, minimum exposure duration, extent of exposure, and combined exposure or latent period. The results from the NCI cohort and the World Trade Center Health Program suggest latent periods of approximately 15 years for NPC and 2 years for LHM, based on statistical modeling and epidemiological studies [19, 46, 66]. In addition, the EU’s “Information notices on occupational diseases: a guide to diagnosis” suggest a 10-year latent period for NPC and 6 months for the minimum exposure duration, despite the absence of definitive scientific evidence [63]. The results from NCI cohort studies also suggest that peak exposures of ≥4.0 ppm were important for LHM and Hodgkin lymphoma [20, 47]. Finally, there is a considerable risk of combined formaldehyde exposure, as the known environmental risk factors for NPC include Epstein-Barr virus infection, consuming salted fish and reserved food spicy food, chronic ear-nose-and-throat conditions, and occupational exposures (e.g., wood dust, industrial heat or combustion products, cotton dust, and solvents, such as phenoxy acid and chlorophenol). These factors must also be considered when determining whether cases are eligible for compensation [67, 68]. Moreover, exposure to benzene, 1,3-butadiene, or ethylene oxide is also an important risk for LHM [69].

## Discussion

The IARC and NTP have classified formaldehyde as a definite human carcinogen, although the US EPA, ACGIH, and EU CLP disagree with this classification [4–7]. A few countries, including South Korea, have also listed formaldehyde as an occupational carcinogen [2, 9–11] because of the relatively low risks of NPC or LHM in meta-analyses and cohort studies (vs. other occupational cancers). Furthermore, it is difficult to quantify FORMALDEHYDE exposure and NPC has a very low incidence (approximately 1/100,000 population) [70]. However, there is sufficient epidemiological evidence to confirm associations with LHM and Hodgkin lymphoma, especially in terms of peak exposure, based on a recent update of the NCI cohort, three recent meta-analyses, and a nested case-control study of embalmers [4, 47].

In South Korea, the peak exposure in various industries was 2.70–14.8 ppm, and the TWA exposure level was 1.0–62.5 ppm in work-environment measurements. Thus, the risk of NPC or LHM could be increased among South Korean pathologists, anatomy students, and furniture workers with a peak exposure of ≥4 ppm [13–15, 17]. In most regions, the age-standardized incidence of NPC among men and women is < 1/100,000 person-years [70]. However, dramatically elevated rates are observed in the Cantonese population of southern China (including Hong Kong) [68]. These regional differences may be related to environmental risk factors, such as Epstein-Barr virus infection, and/or diet [67]. Thus, we suggest that both occupational exposure and environmental risk factors should be considered in the process of approving LHM cases for workers’ compensation.

The present study provided a review of the recent epidemiological evidence regarding the relationships between formaldehyde exposure and NPC or LHM, as well as a discussion regarding factors that could influence the recognition of formaldehyde-related cancers as occupational cancers. However, there is insufficient data regarding peak exposure levels and average exposure levels in various South Korean industries and jobs. Thus, additional studies are needed to help develop compensation policy and achieve scientific consensus.

## Conclusion

We identified causal relationships and significant dose-response relationships between formaldehyde exposure and NPC, all LHM, and Hodgkin lymphoma. Furthermore, it appears that peak exposure is the most relevant factor when considering whether to officially recognize formaldehyde-related occupational cancers. Therefore, it is important to control formaldehyde exposure to protect workers and prevent them from developing NPC or LHM.

## Abbreviations

ACGIH: American Conference of Governmental Industrial Hygienists
EPA: Environmental Protection Agency
EU CLP: European Union Occupational Disease Classification, Labelling and Packaging of Substances and Mixtures
IARC: International Agency for Research on Cancer
ILO: International Labor Organization
LHM: lymphohematopoietic malignancies
NCI: National Cancer Institute
NPC: nasopharyngeal cancer
NTP: National Toxicology Program
TLV-TWA: threshold limit value-time weighted average

## References

[1] Song J, Kim I, Choi BS (2014). The scope and specific criteria of compensation for occupational diseases in Korea. J Korean Med Sci.

[2] Kim I, Kim EA, Kim JY (2014). Compensation for occupational cancer. J Korean Med Sci.

[3] IARC (2006). Formaldehyde, 2-butoxyethanol and 1-tert-butoxypropan-2-ol. Monogr Eval Carcinog Risks Hum.

[4] IARC (2012). A review of human carcinogens: chemical agents and related occupations: formadehyde. Monogr Eval Carcinog Risks Hum.

[5] EPA (2010). IRIS toxicological review of formaldehyde-inhalation assessment (external review of draft).

[6] Eurpoean Composites Industry Association (2014). New classification for formaldehyde and styrene: make sure that your classification, label and safety data sheet (SDS) are correct!, EU, 6th adaptation to technical progress (ATP) to the classification, labelling and packaging of substances and mixtures (CLP) regulation. Belgium.

[7] American Conference of Govermantal Industrial Hygienists (2009). Occupational exposure limits for formaldehyde.

[8] International Labor Organization (2010). Identification and recognition of occupational diseases: criteria for incorporating diseases in the ILO list of occupational diseases.

[9] Kim EA, Kang SK. Historical review of the list of occupational diseases recommended by the International Labour Organization. Ann Occup Environ Med. 2013; 25: 14. 10.1186/2052-4374-25-14 PMC3923370 24472440

[10] European commission. Report on the current situation in relation to occupational diseases’ systems in EU Member States and EFTA/EEA countries, in particular relative to Commission Recommendation 2003/670/EC concerning the European Schedule of Occupational Diseases and gathering of data on relevant related aspects. 2013.

[11] Workers’ compensation act, cf. consolidated act no. 848 of September 9, 2009. Appendix 1. List of occupational diseases reported on or after January 1, 2005. Denmark; 2009.

[12] kim E-A, Yoo K, Ko K. The Prevalence of Occupational Carcinogen Exposure in Korean Workers (I). Incheon, Korea: Occupational Safety and Health Research Institute; 2011.

[13] Park JY, Zong MS (1998). A study on woker-expoure to formaldehyde in some histological laboratories of hospitals. Korean Ind Hyg Assoc J.

[14] Park SY, Kim CY, Kim JY, Sakong J (2016). The health effects of formaldehyde during an anatomy dissection course. Korean J Occup Environ Med.

[15] Kim JH, Kim DJ, Kim H (2009). A study on exposure-worker to formaldehyde in the endoscopy unit of hospitals. J Korean Soc Occup Environ Hyg.

[16] Lee N, Lee B, Jeong S, Yi GY, Shin J (2012). Work environments and exposure to hazardous substances in Korean tire manufacturing. Saf Health Work.

[17] Yoo K, Lee MY (2014). Identification of process generating formaldehyde in a furniture manufacturer. Analytical Science & Technology.

[18] Gu D, Lee C, Lee J, Lee S, Yun S, Han A, Kim H, Park Y, Jeong S, Moon C (2014). Exposure to formaldehyde of ambulatory care nurses in university hospital. J Korean Soc Occup Environ Hyg..

[19] Hauptmann M, Lubin JH, Stewart PA (2004). Mortality from solid cancers among workers in formaldehyde industries. Am J Epidemiol.

[20] Freeman LEB, Blair A, Lubin JH, Stewart PA, Hayes RB, Hoover RN, Hauptmann M (2013). Mortality from solid tumors among Workers in Formaldehyde Industries: an update of the NCI cohort. Am J Ind Med.

[21] Marsh GM, Youk AO (2005). Reevaluation of mortality risks from nasopharyngeal cancer in the formaldehyde cohort study of the National Cancer Institute. Regul Toxicol Pharmacol.

[22] Marsh GM, Youk AO, Morfeld P (2007). Mis-specified and non-robust mortality risk models for nasopharyngeal cancer in the National Cancer Institute formaldehyde worker cohort study. Regul Toxicol Pharmacol.

[23] Coggon D, Harris EC, Poole J, Palmer KT (2003). Extended follow-up of a cohort of british chemical workers exposed to formaldehyde. J Natl Cancer Inst.

[24] Coggon D, Ntani G, Harris EC, Palmer KT (2014). Upper airway cancer, myeloid leukemia, and other cancers in a cohort of British chemical workers exposed to formaldehyde. Am J Epidemiol.

[25] Pinkerton LE, Hein MJ, Stayner LT (2004). Mortality among a cohort of garment workers exposed to formaldehyde: an update. Occup Environ Med.

[26] Meyers AR, Pinkerton LE, Hein MJ (2013). Cohort mortality study of garment industry workers exposed to formaldehyde: update and internal comparisons. Am J Ind Med.

[27] Siew SS, Kauppinen T, Kyyrönen P, Heikkilä P, Pukkala E (2012). Occupational exposure to wood dust and formaldehyde and risk of nasal, nasopharyngeal, and lung cancer among Finnish men. Cancer Management Reasearch.

[28] Pira E, Romano C, Federica V, La Vecchia C (2014). Mortality from lymphohematopoietic neoplasms and other causes in a cohort of laminated plastic workers exposed to formaldehyde. Cancer Causes Control.

[29] Roush GC, Walrath J, Stayner LT (1987). Nasopharyngeal cancer, sinonasal cancer, and occupations related to formaldehyde: a case-control study. J Natl Cancer Inst.

[30] West S, Hildesheim A (1993). Dosemeci M (1993). Non-viral risk factors for nasopharyngeal carcinoma in the Philippines: results from a case-control study. Int J Cancer.

[31] Vaughan TL, Stewart PA, Teschke K (2000). Occupational exposure to formaldehyde and wood dust and nasopharyngeal carcinoma. Occup Environ Med.

[32] Olsen JH, Jensen SP, Hink M (1984). Occupational formaldehyde exposure and increased nasal cancer risk in man. Int J Cancer.

[33] Vaughan TL, Strader C, Davis S, Daling JR (1986). Formaldehyde and cancers of the pharynx, sinus and nasal cavity: I. Occupational exposures. Int J Cancer.

[34] Armstrong RW, Imrey PB, Lye MS (2000). Nasopharyngeal carcinoma in Malaysian Chinese: occupational exposures to particles, formaldehyde and heat. Int J Epidemiol.

[35] Hildesheim A, Dosemeci M, Chan CC (2001). Occupational exposure to wood, formaldehyde, and solvents and risk of nasopharyngeal carcinoma. Cancer Epidemiol Biomark Prev.

[36] Collins JJ, Acquavella JF, Esmen NA (1997). An updated meta-analysis of formaldehyde exposure and upper respiratory tract cancers. J Occup Environ Med.

[37] Bosetti C, JK ML, Tarone RE (2008). Formaldehyde and cancer risk: a quantitative review of cohort studies through 2006. Ann Oncol.

[38] Bachand AM, Mundt KA, Mundt DJ, Montgomery RR (2010). Epidemiological studies of formaldehyde exposure and risk of leukemia and nasopharyngeal cancer: a meta-analysis. Crit Rev Toxicol.

[39] Walrath J & Fraumeni JF Jr. Mortality patterns among embalmers. Int J Cancer 1983, 31: 407–411. 10.1002/ijc.2910310403 6832852

[40] Walrath J, Fraumeni JF (1984). Cancer and other causes of death among embalmers. Cancer Res.

[41] Levine RJ, Andjelkovich DA, Shaw LK (1984). The mortality of Ontario undertakers and a review of formaldehyde-related mortality studies. J Occup Med.

[42] Stroup NE, Blair A, Brain EGE (1986). Cancer and other causes of death in anatomists. J Natl Cancer Inst.

[43] Logue JN, Barrick MK, Jessup GL (1986). Mortality of radiologists and pathologists in the radiation registry of physicians. J Occup Med.

[44] Hayes RB, Blair A, Stewart PA (1990). Mortality of U.S. embalmers and funeral directors. Am J Ind Med.

[45] Hall A, Harrington JM, Aw TC (1991). Mortality study of British pathologists. Am J Ind Med.

[46] Hauptmann M, Lubin JH, Stewart PA (2003). Mortality from lymphohematopoietic malignancies among workers in formaldehyde industries. J Natl Cancer Inst.

[47] LEB F, Blair A, Lubin JH (2009). Mortality from lymphohematopoietic malignancies among workers in formaldehyde industries: the National Cancer Institute cohort. J Natl Cancer Inst.

[48] Checkoway H, Dell LD, Boffetta P, Gallagher AE, Crawford L, Lees PS, Mundt KA (2015). Formaldehyde exposure and mortality risks from acute myeloid leukemia and other Lymphohematopoietic malignancies in the US National Cancer Institute cohort study of Workers in Formaldehyde Industries. J Occup Env Med.

[49] Linos A, Blair A, Cantor KP (1990). Leukemia and non-Hodgkin’s lymphoma among embalmers and funeral directors. J Natl Cancer Inst.

[50] Partanen T, Kauppinen T, Luukkonen R (1993). Malignant lymphomas and leukemias, and exposures in the wood industry: an industry-based case-referent study. Int Arch Occup Environ Health.

[51] Blair A, Zheng T, Linos A (2001). Occupation and leukemia: a population-based case-control study in Iowa and Minnesota. Am J Ind Med.

[52] Hauptmann M, Stewart PA, Lubin JH (2009). Mortality from lymphohematopoietic malignancies and brain cancer among embalmers exposed to formaldehyde. J Natl Cancer Inst.

[53] Collins JJ, Lineker GA (2004). A review and meta-analysis of formaldehyde exposure and leukemia. Regul Toxicol Pharmacol.

[54] Zhang L, Steinmaus C, Eastmond DA (2009). Formaldehyde exposure and leukemia: a new meta-analysis and potential mechanisms. Mutat Res.

[55] Schwilk L, Zhang L, Smith MT, Smith AH, Steinmaus C (2010). Formaldehyde and leukemia: am updated meta-analysis and evaluation of bias. J Occup Environ Med.

[56] Checkoway H, Boffetta P, Mundt DJ, Mundt KA (2012). Critical review and synthesis of the epidemiologic evidence on formaldehyde exposure and risk of leukemia and other lymphohematopoietic malignancies. Cancer Causes Control.

[57] Morfeld P (2013). Formaldehyde and leukemia: missing evidence!. Cancer Causes Control.

[58] Georgieva AV, Kimbell JS, Schlosser PM (2003). A distributed-parameter model for formaldehyde uptake and disposition in the rat nasal lining. Inhal Toxicol.

[59] Zhang L, Tang X, Rothman N (2010). Occupational exposure to formaldehyde, hematotoxicity, and leukemia-specific chromosome changes in cultured myeloid progenitor cells. Cancer Epidemiol Biomark Prev.

[60] Lan Q (2015). Chromosome-wide aneuploidy study of cultured circulating myeloid progenitor cells from workers occupationally exposed to formaldehyde. Carcinogenesis.

[61] National Toxicology Program. Report on carcinogens, Thirteenth Edition. US Department of Health and Human Services, Public Health Service, National Toxicology Program. 2016. Available at: https://ntp.niehs.nih.gov/pubhealth/roc/index-1.html#toc1.

[62] Finnish Institute of Occupational Health. Memorandum from the Occupational Cancer Working Group 2013. Finnish institute of occupational health.Helsinki.

[63] European commission. Information notices on occupational diseases: a guide to diagnosis. Office for Official Publications of the European Communities. Luxembourg. 2009:2009.

[64] Jung PK, Kim I, Park I, Kim C, Kim EA, Roh J (2014). A case of mutiple myeloma in a poultry worker. Ann Occup Environ Med.

[65] Choi M, Yoo KM, Park CY, Park HH, Kim HR. A case of myelodysplatic syndrome in a worker assembling medium density fibreboard. Procedding of the 53th conference of Korean society of occupational and environmental medicine. P415. (Korean).

[66] Howard J. Minimum Latency & Type of Cancer. Replaces administrator’s white paper on minimum latency & types of cancer. Centers for disease control and prevention. 2013. http://www.cdc.gov/wtc/pdfs/wtchpminlatcancer2013-05-01.pdf.

[67] Chang ET, Adami HO (2006). The enigmatic epidemiology of nasopharyngeal carcinoma. Cancer Epidemiol Biomark Prev.

[68] Jia WH, Qin HD (2012). Non-viral environmental risk factors for nasopharyngeal carcinoma: a systematic review. In Semin Cancer Biol.

[69] Eastmond DA, Keshava N, Sonawane B (2014). Lymphohematopoietic cancers induced by chemicals and other agents and their implications for risk evaluation: an overview. Mutarion Research.

[70] Torre LA, Bray F, Siegel RL, Ferlay J, Lortet-Tieulent J, Jemal A (2015). Global cancer statistics, 2012. CA Cancer J Clin.

